# Differential diagnosis of dizziness after a sports-related concussion based on descriptors and triggers: an observational study

**DOI:** 10.1186/s40621-015-0055-2

**Published:** 2015-09-17

**Authors:** Jennifer C. Reneker, Vinay Cheruvu, Jingzhen Yang, Chad E. Cook, Mark A. James, M. Clay Moughiman, Joseph A. Congeni

**Affiliations:** 1 Department of Biostatistics, Environmental Health Sciences, and Epidemiology, College of Public Health, Kent State University, Kent, OH USA; 2 Physical Therapy Program, Division of Health Sciences, Walsh University, North Canton, OH USA; 3 Center for Injury Research and Policy, The Research Institute at Nationwide Children’s Hospital, Department Of Pediatrics, College of Medicine, The Ohio State University, Columbus, OH USA; 4 Division of Physical Therapy, Department of Orthopaedics, Duke University, Durham, NC USA; 5 Physical Therapy Service, Community Based Outpatient Services, Louis Stokes Cleveland VA Medical Center, Cleveland, OH USA; 6 Center for Orthopedics and Sports Medicine, Akron Children’s Hospital, Considine Professional Building, Akron, OH USA

## Abstract

**Background:**

Dizziness is often reported after a sports-related concussion. Forces experienced at the time of the concussion can cause an injury to multiple anatomical areas, including the central nervous system, the vestibular system, and the cervical spine, each of which is sufficient to cause dizziness. Medical professionals routinely use the subjective history to develop hypotheses about what may be causing a patient’s dizziness. No previous studies have attempted to differentiate the source of the dizziness through precise patient descriptors or the triggers of dizziness.

**Methods:**

A structured symptom questionnaire was developed through purposive exploration of relevant literature for common dizziness quality descriptors and triggers. This questionnaire was used to interview a sample of 86 adolescent athletes (12–19 years of age) with a sports-related concussion between August 2013 and April 2014. Exploratory Latent Class Analysis was used to uncover latent constructs within the 15 dizziness descriptors and 11 dizziness triggers. The covariates sex, attention deficit hyperactivity disorder, and number of days between the concussion and the assessment were added to the model to estimate if these variables influenced class membership probabilities.

**Results:**

Thirty-two (36 %) of the patients interviewed did not report a complaint of dizziness but did affirm one or more of the other descriptors. Three classes of dizziness based on dizziness quality descriptors and three classes based on dizziness triggers were identified by the analysis. Neither the classes of descriptors nor the classes of triggers enabled differentiation based on anatomical etiology of the dizziness.

**Conclusions:**

Patient description of dizziness is limited in its ability to assist in differential diagnosis based on anatomical location for athletes with concussion. This may be because more than one area is contributing to the dizziness or because concussed adolescents have difficulty describing the way that they feel. In this case, solely relying on the patient to provide a description of dizziness to develop the formation of hypotheses and lead the direction of objective tests is inappropriate. If the scope of the objective assessment is limited by the patient description of dizziness, it is likely that areas of dysfunction may be overlooked.

## Background

Sports-related concussion (SRC) is a major public health concern for youth participating in contact sports. According to the Centers for Disease Control and Prevention (CDC), between 2009 and 2011, the number of reported concussions increased by nearly 100,000 in the 10–19-year-old age group (Faul et al. 2010). The most commonly related symptoms after a concussion are headache, dizziness (Alsalaheen et al. 2010; Makdissi et al. 2010), postural disturbances/balance problems (Alsalaheen et al. 2010), and neck pain. Second only to headache, dizziness is present in 43 to 81 % of concussions (Lau et al. 2011; Duhaime et al. 2012; Alsalaheen et al. 2010).

Since a concussion is caused by a direct blow to the head, face, neck, or elsewhere on the body with an “impulsive” force transmitted to the head (Lanza et al. 2007), it is possible that a common complaint of dizziness is derived from different underlying mechanisms. In the presence of SRC, the sensation of dizziness could be caused from functional disturbances of sensory processing by the central nervous system (Guskiewicz and Register-Mihalik 2011; Lovell et al. 2006). Additionally, the biomechanical forces experienced by an athlete concomitant to causing the concussion are also capable of disrupting the peripheral vestibular system as well as the cervical spine. Injury to or dysfunction of any of these areas is sufficient to cause a complaint of dizziness (Guskiewicz and Register-Mihalik 2011). In the presence of concussion and dizziness, the challenge of identifying the underlying cause and establishing an appropriate diagnosis becomes complicated.

Currently, as standard practice, when performing an examination on a patient with a report of dizziness, a directed subjective history is considered as the “single most important element in reaching a correct diagnosis (Al Saif and Alsenany 2015).” Patient report is used to assist in determining what may be causing a patient’s dizziness (Herdman and Clendaniel 2014; Landel 2010). Patient responses can then be used to drive the objective portion of the examination (Chan 2009; Wrisley et al. 2000). It is believed that gaining insight into an exact dizziness description as well as the tempo and triggers, especially in an outpatient setting, is useful and necessary to guide the objective assessment (Herdman and Clendaniel 2014; Landel 2010; Chan 2009; Kristjansson and Treleaven 2009). For medical professionals, differentiating the etiology dizziness at the time of the assessment is necessary in order to deliver appropriate treatment to the targeted area.

Dizziness, within the general population, is generally used as a symptom descriptor for three main abnormal sensations: true vertigo (feeling of spinning), light-headedness (pre-syncope), and disequilibrium (feeling off-balance) (Herdman and Clendaniel 2014; Landel 2013). Patients may report a general sense of dizziness accompanied with a wide variety of other descriptions to distinguish their experience (Landel 2013; Chan 2009). In addition, there are a variety of triggers (or provoking activities) that are commonly associated with dizziness, often including eye, head, or body movements or environment triggers (e.g., objects moving quickly within vision of the patient) (Landel 2013; Herdman and Clendaniel 2014). Given the diversity of sensations and activities that can converge on a patient report of “I feel dizzy,” it is impossible to know precisely what is being experienced without additional questioning to further elucidate the meaning and triggers of “dizzy.”

Dizziness from a central origin is often associated with descriptions of disequilibrium, motion sensitivity, and nausea and is often triggered by sensory processing of mismatched afferent inputs during movement (Herdman and Clendaniel 2014). Peripheral vestibular dizziness is most often associated with a report of the sensation of spinning, disequilibrium, and nausea and may be accompanied by the illusion of visual motion (oscillopsia). Triggers of vestibular dizziness most commonly include movements of the head (Herdman and Clendaniel 2014). Dizziness derived from the cervical spine is generally regarded as a diagnosis of exclusion (Landel 2013; Wrisley et al. 2000) and is often regarded as non-specific dizziness (Landel 2013; Kristjansson and Treleaven 2009; Treleaven 2008). Experts in cervicogenic dizziness have identified common patient descriptions for this type of dizziness that include imbalance or unsteadiness (disequilibrium) (Wrisley et al. 2000; Landel 2013; Al-Saif et al. 2012; Herdman and Clendaniel 2014), light-headedness (Herdman and Clendaniel 2014; Landel 2013), nausea (Al-Saif et al. 2012), floating, spacey, feeling “off,” and difficulty with concentration or focus (Landel 2010). It is also expected that these sensations will be triggered by head/neck position (Wrisley et al. 2000; Landel 2013).

There are no published studies that have attempted to differentiate sub-types of concussion-derived dizziness based on patient presentation and report of the quality descriptors or the triggers (activities that provoke) of dizziness. Therefore, the purpose of this study was to test the ability of a structured symptom questionnaire to differentiate the patient complaint of dizziness in adolescent athletes with SRC. It was hypothesized that within the dizziness descriptors and the dizziness triggers (separately), three distinct types of dizziness would be identified: dizziness associated with central nervous system disturbance, cervicogenic dizziness, and dizziness derived from peripheral vestibular dysfunction.

## Methods

### Ethics, consent, and permissions

This was an observational study of the natural history of SRC, incorporating a structured patient questionnaire. This study was approved by the Institutional Review Boards at Akron Children’s Hospital and Kent State University. Participant consent (if older than 18 years of age) or parental consent and participant assent (if younger than 18 years of age) were obtained prior to enrollment. The study population included adolescent athletes diagnosed with SRC who were seen at the Sports Medicine Center at Akron Children’s Hospital for an initial assessment between August 2013 and April 2014. There were 111 participants enrolled with a concussion, of which 86 endorsed one or more of the dizziness quality descriptors and were included in the analyses.

Based on convenience, participants were invited to participate in the study if they were between 12 and 19 years of age. For this study, a diagnosis of SRC required that the concussion occurred during participation in a sport activity, which included participation in a competition or recreational activity, including formally organized contact school sports, participation in gym class, skiing, running, skateboarding, and other informal recreation activities (Noble and Hesdorffer 2013). Sport activity did not include participation in recreational activities on a motorized vehicle (e.g., motor vehicle accidents or all-terrain vehicle accidents). Student athletes with pre-morbid conditions such as attention deficit hyperactivity disorder (ADHD) or prior concussion history were included in this study.

Measurement of the sensation of dizziness experienced by each participant was recorded with a symptom questionnaire including 15 quality descriptors of dizziness and 11 triggers of dizziness with dichotomous response (answered yes/no). This instrument was developed after searching peer reviewed and gray literature to find the most common patient descriptors and triggers that may implicate different etiologies of dizziness (potentially differentiating the anatomical areas contributing to dizziness) (Al-Saif et al. 2012; Lau et al. 2011; Wrisley et al. 2000; Kristjansson and Treleaven 2009; Herdman and Clendaniel 2014; Landel 2013). Clinician and research experts (*n* = 11) were asked to comment on the comprehensiveness of the tool for use in differential assessment of dizziness (content validity). Face validity, with both non-concussed (*n* = 7) and concussed athletes (*n* = 10), was established during the development of the symptom questionnaire.

The symptom questionnaire was administered during the initial clinical visit following the concussion. Trained study staff read the questions to each participant and recorded the answer for each question. All questions were read exactly as printed with no additional clarification of wording or patient responses. If clarification was requested or if the athlete did not know the answer, the item was recorded as unknown. Data on participant characteristics including sex, birth date, the sport where the concussion occurred, previous concussions, and pre-morbid ADHD were also collected.

### Statistical analysis

An exploratory analysis was conducted to determine if a hidden structure was present within the dizziness quality descriptors and separately for the dizziness triggers using Latent Class Analysis (LCA). LCA is a statistical method used to uncover the underlying structure (latent constructs) of the relatively large set of binomial variables (i.e., the items in the structured questionnaire). With LCA, the probabilities of class membership as well as the probabilities of item response (conditional on class membership) are estimated based on maximum likelihood estimation (Lanza et al. 2007). The three key factors used to determine whether the LCA model is the most optimal to describe the behavior of the data were as follows: the primary hypothesis (i.e., that three classes would be identified by the model), the fit statistics obtained by each model (Akaike’s Information Criterion), and the influence relevant covariates had in the final model.

After considering the fit statistics, the latent classes obtained were evaluated qualitatively for consistency in theme and labeled appropriately, potentially defining different sub-types of dizziness (based separately on the quality descriptors and the triggers). The literature used to develop the questionnaire was again utilized to evaluate the classes and determine if the item response patterns within the classes adequately described the expected probability of dizziness quality descriptors for central, vestibular, and cervical types of dizziness (Herdman and Clendaniel 2014; Landel 2013; Wrisley et al. 2000; Al-Saif et al. 2012; Lau et al. 2011; Guskiewicz and Register-Mihalik 2011; Lovell et al. 2006). This process was repeated for the dizziness triggers. Finally, relevant covariates, which may have influenced class membership probabilities, were added to the model one at a time for univariate analysis. All statistical analyses were completed using SAS, version 9.3.

## Results

Descriptive statistics of the subjects are presented in Table 1. Within the sample, 52 (60.5 %) participants were males and ranged in age from 12.24 to 19.63 years with a mean of 15.07 (SD = 1.58). The participants were interviewed with the structured symptom questionnaire a mean of 8.8 days after the time of the initial injury (SD = 10.7 and median = 6 days; interquartile range Q1 = 4; Q3 = 11), with a range of 1–89 days. Within the sample, 8 participants reported a personal history of ADHD and 27 reported at least 1 prior concussion. A majority of the participants sustained their concussion during participation in football (*n* = 32), basketball (*n* = 14), and soccer (*n* = 12). Regarding dizziness, 54 (63 %) of the participants affirmed the sensation of dizziness (Table 2). Thirty-two participants did not report the sensation of dizziness but did affirm one or more of the other 14 dizziness descriptors.

**Table 1:** Descriptive statistics of the sample (*n* = 86)

Variables	Mean (SD)	Range
Age in years	15.1 (1.6)	12.2–19.6
Number of days between the date of concussion and assessment	8.8 (10.7) Median = 6	1–89
	Frequency	Percent
Sex (male)	52	60
History of ADHD	8	9
Previously diagnosed with a concussion	27	31
Number of previously diagnosed concussions		
1	19	22
2	8	9
Sport where concussion was sustained^a^		
Football	32	38
Soccer	12	14
Baseball/softball	1	1
Hockey	1	1
Cheerleading	3	4
Basketball	14	16
Lacrosse	1	1
Volleyball	2	2
Wrestling	4	5
Swimming	2	2
Rugby	1	1
Other sport activity	12	14

^a^One not reported

**Table 2:** Frequency of dizziness descriptor and probability of endorsement of each item given latent class membership

		Probability of “yes” response given latent class (SE)		
Question asked of the participants	Yes, *n* (%)	High symptoms	Medium symptoms	Low symptoms
Are you dizzy?	54 (63)	.99 (02)	.63 (.08)	.27 (.10)
Do you feel like the room is spinning?	5 (6)	–	–	–
Do you feel like stationary objects are moving?	9 (10)	.39 (.12)	.00 (.00)	.04 (.04)
Do you have imbalance or unsteadiness when standing (like you might fall or are swaying)?	43 (50)	.98 (.04)	.40 (.09)	.24 (.10)
When you turn your head, does it seem like your eyes do not keep up?^a^	25 (30)	.41 (.12)	.39 (.08)	.01 (.02)
Do you feel like you are floating?	3 (3)	–	–	–
Do you feel spacey?	26 (30)	.39 (.12)	.40 (.08)	.01 (.04)
Do you feel like something is just “off”?	64 (74)	.92 (.07)	.91 (.05)	.24 (.10)
Do you have a “fuzzy head”?	37 (43)	.83 (.09)	.42 (.08)	.06 (.06)
Do you feel light-headed (like you might pass out)?	26 (30)	.68 (.13)	.25 (.07)	.05 (.06)
Do you have difficulty concentrating or focusing?	67 (78)	.96 (.05)	.80 (.07)	.56 (.11)
Do you have a “swimming” sensation in your head?^b^	5 (6)	–	–	–
Do you have motion sickness?	18 (21)	.47 (.12)	.14 (.06)	.10 (.07)
Do you feel nauseous (like you might throw up)?	22 (26)	.71 (.13)	.16 (.06)	.00 (.01)
Is the way you feel difficult to describe?	67 (78)	.94 (.05)	.88 (.05)	.42 (.12)

Three items were removed from the final model because the item response probabilities were not distinguishable between classes (i.e., the differences in item response probability between classes was less than 15 %)

^a^Missing values

^b^2 missing values

### LCA: dizziness quality descriptors

The three-class LCA with the 15 dizziness quality descriptors revealed a poorly specified model. Rerun as a two-class model, the Akaike’s Information Criterion (AIC) increased (indicating worse fit). A visual analysis of the two- and the three-class models was carried out to determine if any of the items should be removed from the analysis to decrease the complexity of the model. Because of low “yes” response in the items spinning, floating, and swimming (<6 % in all cases), the probabilities of item response was very low (<17 %) in all three classes. For this reason, these items were removed from the analysis, leaving 12 items in the final analysis.

The three-class LCA with the 12 remaining items revealed a better model specification, with a large drop in AIC. The two-class model with the 12 items resulted in an increase in the AIC so it was determined that the three-class model with the 12 descriptors was the best fitting model for these data (Table 4). Within this final model, the probabilities of class membership (for the participants in the sample) were 24 % for the first class, 51 % for the second class, and 25 % for the third class. With the addition of covariates to the final model, it was found that none of the covariates (sex, ADHD, and the number of days between the assessment and the date of the concussion) were significant determinants of class membership (Table 5).

### LCA: dizziness triggers

A comparison was made between the three-class LCA and the two-class LCA with the 11 dizziness triggers (Table 3). Based on the fit statistics obtained, it was determined that the three-class model provided a better fit for these data. Upon visual analysis of the response probabilities between the classes, it was determined to keep all 11 items within the final model.

**Table 3:** Frequency of dizziness trigger and probability of endorsement of each item given latent class membership

		Probability of “yes” response given latent class (SE)		
Question asked of the participants	Yes, *n* (%)	Visual triggers	Visual and movement triggers	Undefined triggers
Sitting still with no movement	11 (13)	.04 (.05)	.12 (.06)	.20 (.08)
Looking at a computer^a^	53 (65)	.94 (.06)	.99 (.02)	.02 (.05)
Watching television^b^	43 (51)	.85 (.09)	.74 (.08)	.01 (.02)
Reading^c^	50 (63)	.54 (.12)	.92 (.06)	.35 (.09)
With your eyes closed	13 (15)	.00 (.01)	.31 (.08)	.07 (.05)
Looking up towards the sky^d^	23 (28)	.05 (.06)	.54 (.09)	.14 (.07)
Bending down towards the floor	34 (40)	.01 (.02)	.76 (.09)	.28 (.08)
Turn your head left and right^d^	21 (25)	.06 (.06)	.45 (.09)	.17 (.07)
Roll over in bed	47 (55)	.21 (.12)	.84 (.06)	.46 (.09)
Ride in a car	30 (35)	.19 (.09)	.52 (.09)	.25 (.08)
When you move quickly or when things move quickly around you	70 (82)	.77 (.10)	.91 (.05)	.76 (.08)

^a^3 missing values

^b^2 missing values

^c^5 missing values

^d^1 missing values

Within the final three-class model, the probabilities of class membership for the sample were 24 % for the first class, 41 % for the second class, and 35 % for the third class. With the univariate addition of covariates, it was determined that ADHD and the number of days between the concussion and the date of the assessment were not significant determinants of class membership (Table 4). Sex was a significant predictor of class membership, with males having a lower odds (OR = .24; 95 % CI = .07, .88) of membership in the undefined triggers class (Table 5).

**Table 4:** Comparison of baseline models for dizziness quality descriptors and triggers

	Number of classes	Number of items	AIC
Quality descriptors	3	15	497.4
	*3*	*12*	*393.7*
	2	15	501.9
	2	12	400.9
Triggers	*3*	*11*	*309.6*
	2	11	338.0

Values in italics indicate the chosen model

*AIC* Akaike’s Information Criterion

**Table 5:** Baseline model membership probability and odds ratios for class membership based on relevant covariates

Baseline model class membership probabilities for dizziness quality descriptors	Class name		
	24 %	51 %	25 %
	High symptoms	Medium symptoms	Low symptoms
Adjusted model ORs of class membership			
Male	Reference	1.2 (.26, 5.3)	2.4 (.63, 9.5)
ADHD	Reference	.64 (.04, 9.65)	4.7 (.57, 39.3)
Number of days to assessment	Reference	1.02 (.95, 1.10)	1.05 (.98, 1.13)
Baseline model class membership probabilities for dizziness triggers	Class name		
	24 %	41 %	35 %
	Visual	Visual and movement	Other triggers
Adjusted model ORs of class membership			
Male	Reference	2.0 (.51, 8.4)	*.24 (.07, .88)*
ADHD	Reference	1.89 (.33, 10.7)	.31 (.02, 3.46)
Number of days to assessment	Reference	.98 (.94, 1.03)	1.00 (.96, 1.04)

Values in italics indicate significant findings

## Discussion

The purpose of this study was to determine if it was possible to differentiate the patient complaint of dizziness in adolescent athletes with SRC through the use of a structured symptom questionnaire. After model assessment for both analyses (descriptors and triggers), three-class models were determined to have the best fit for these data. Despite this, upon qualitative assessment, the classes for both the descriptors and the triggers were unable to be meaningfully labeled based on the anatomical derivation of the dizziness (central, peripheral vestibular, and cervical) as hypothesized. For the descriptors, the classes were labeled according to the number of items with high probabilities of yes responses (high number of descriptors, medium number of descriptors, and low number of descriptors). For the triggers, the classes were labeled according to the type of triggers most often associated with the provocation of the dizziness (visual triggers, visual and movement triggers, and undefined triggers). In this analysis, sex was associated with class membership, with males demonstrating significantly lower odds of membership in the undefined triggers class.

Given the results of the analysis on this sample, by themselves, the classes obtained and the labels applied to the classes for the dizziness quality descriptors appear to offer little to no anatomical differentiating information. This is potentially because there is no discernable pattern of dizziness within the quality descriptors or because there are more than three classes of dizziness and this study was not powered to identify additional classes. It is also likely that in the presence of SRC, with differing severities, more than one area is contributing to the dizziness (cervicogenic and central and peripheral vestibular) and thusly differentiating between these types is not possible.

Within the descriptors of dizziness, an unexpected finding was that 36 % of the participants did not endorse the complaint of dizziness but did endorse one of the other 14 descriptors of dizziness. It was thought that the prevalence of dizziness would be 100 % in the sample, but this was not the case. Given that in a clinical setting, “dizziness” is usually the starting point for differential questions (Newman-Toker et al. 2007) (i.e., What do you mean by “dizzy”?), it is important to note that although abnormal sensations associated with the perception of movement (self or environmental) were present in this sample, this sense was not perceived as dizziness by over one third of the participants. This finding indicates that dizziness may not be the prevailing descriptor for this abnormal sense in adolescents with concussion. This needs to be considered when obtaining symptom report because commonly used self-report symptom scales (such as the Post-Concussion Scale and the Sports Concussion Assessment Tool 3) only include dizziness, but not the other descriptors of dizziness. A true functional/perceptual injury may be completely missed in those athletes who do not equate their abnormal sense as “dizziness.”

Another point of interest was that spinning (vertigo), which is the most often associated complaint with vestibular system etiologies of dizziness (Herdman and Clendaniel 2014; Newman-Toker et al. 2007), was only endorsed by five of the participants and, because of the lack of differentiation between classes, was not included in the final model. For the vast majority of participants in this sample (*n* = 81 with no report of spinning), this could indicate one of two things: the vestibular system is contributing to their dizziness but these athletes are not experiencing spinning or the vestibular system is not contributing to the dizziness. In either case, this would be a substantial deviation from the expected presentation, as the vestibular system is often implicated as a primary source of patient dizziness after a concussion (Alsalaheen et al. 2010; Alsalaheen et al. 2013).

Within the dizziness triggers, two of the three classes appeared to follow an identifiable pattern but these patterns could not be exclusively named according to the anatomical etiology. The visual trigger class included items generally associated with eye but not body movements (i.e., watching television, looking at a computer screen, and reading) and one item that could include visual processing of environmental motion and/or self-motion (i.e., moving quickly or things moving quickly around you). At first glance, this would appear to indicate centrally induced dizziness. However, the second class (visual and movement class) included all of the visual processing items from the first class and additional items of self-movement (i.e., neck and body), which does not clearly point to one area but could potentially implicate the central nervous system, the vestibular system, and the cervical spine. The final class, undefined triggers, has very low item response probabilities, between 1 and 47 % for all items except moving quickly or when things move quickly around you (73 % “yes” response). This singular item with a relatively high report of causing an exacerbation does not clearly point to one location.

These main findings indicate that the classification of dizziness according to triggers does not permit etiologic differentiation; however, the latent traits identified uncover a common provoking activity. This type of structure is more clinically useful in the approach to a patient than the simple “high, medium, low” classification that we obtained in the analysis of the descriptors. The clinical usefulness of the information provided by a careful exploration of dizziness triggers has been suggested (Newman-Toker et al. 2007; Al Saif and Alsenany 2015; Herdman and Clendaniel 2014; Bisdorff et al. 2009). However, triggers (by themselves) are also limited in clinical usefulness for differential diagnosis (Lawson et al. 2005).

Our findings pointing to the limited usefulness of differential assessment according to patient quality description of dizziness are similar to other research that has been conducted in emergency medicine (Newman-Toker et al. 2007) and in outpatient settings (Treleaven et al. 2008; Zainun et al. 2012). Researchers have demonstrated that dizziness quality descriptors themselves are not useful in differentiation and if over-relied upon, can mislead the direction of objective assessments (Newman-Toker et al. 2007; Treleaven et al. 2008). Although this present study was not carried out in an emergency department, it is possible that because of the acuity of the event in this sample (6 days was the median number of time since the injury), these patients were similarly unable to describe their dizziness in a meaningful manner. Additionally, it has been suggested that if this is the first time these types of sensations have been experienced, patients may have difficulty describing what they are experiencing (Newman-Toker et al. 2007).

Another factor that may influence the usefulness of patient description of dizziness is the emotional sequelae of concussion. Emotional disturbances are commonly present after concussion (Kontos et al. 2012). It has been demonstrated that psychogenic factors (such as anxiety) may factor into the description of dizziness, complicating differential diagnosis based on symptom description (Stone et al. 2005). Although there is potentially an interaction between emotional and physical symptoms in adolescents with SRC, the interplay between them has not been described in concussion literature.

### Limitations

The findings from this research are based on an exploratory analysis with relatively complex models for both the descriptors and the triggers. Within the final chosen model for both analyses, the fit statistics obtained point to a less than well-specified structure of latent traits present within the data. The sample of participants was based on convenience and as such, cannot be confidently generalized to the population of adolescents with a SRC. Content validity was established during the development of the instrument, but there are many other descriptors and triggers that are less common but could have been included and may have offered a different result. Empirical data does not exist in current literature that definitively links specific quality descriptors or triggers to any one sub-type of dizziness, and as such, the quality descriptors and the triggers chosen within the questionnaire were the most commonly presented within published documents. It is possible that individual quality descriptors or triggers of dizziness are associated with specific sub-types of dizziness, but our findings were unable to confirm this. Finally, this study was based on self-reporting, which carries the potential for over- and under-reporting of symptoms (Prince et al. 2008). Additionally, although face validity was established in the development of the questionnaire, it is possible that some of the participants did not understand the quality descriptors or the triggers but answered the question regardless, which would skew the data.

## Conclusions

Our results suggest that patient description of dizziness is somewhat limited in its ability to assist in differential diagnosis based on anatomical location for athletes with concussion. These findings may be due to the multifactorial nature of the injury (with more than one area contributing to the dizziness) or because of difficulty describing the way that they are feeling. In this case, relying on the patient to provide a description of dizziness to develop the formation of hypotheses and lead the direction of objective tests is inappropriate. In light of the findings presented here, healthcare professionals responsible for the medical evaluation and management of individuals with concussion should recognize the potential for missed functional disturbances resulting from the concussion if the patient report of symptoms is over-relied upon. If the scope of the objective assessment is limited by the patient description of dizziness, it is likely that areas of dysfunction may be overlooked. Therefore, for appropriate patient care, it is important for medical professionals to complete a comprehensive physical examination in order to confidently identify all areas contributing to dizziness so that directed and encompassing treatment may be initiated.

We suggest that an exploration of common dizziness triggers may lead to the identification of common movements that provoke the symptom of dizziness (or other descriptions of abnormal sense of self or environmental movement). However, because neither the descriptors nor the triggers clearly point to one anatomical etiology, the objective assessment should be designed to prescriptively examine all of the potential contributing areas, including the central nervous system, the peripheral vestibular system, and the cervical spine. Additional directions of research should explore the existence of an association between specific descriptors or triggers and objective test findings. Other research to explore the type of functional neurological disturbances through appropriate imaging techniques (e.g., diffuse tensor imaging) together with patient symptom description could offer additional insight into the correlation between anatomic injury and patient report. It is believed that continuation of this line of research is imperative to inform appropriate patient management and improve health outcomes after concussion.
